# Investigating proliferation and differentiation capacities of Hanwoo steer myosatellite cells at different passages for developing cell-cultured meat

**DOI:** 10.1038/s41598-023-40800-7

**Published:** 2023-09-20

**Authors:** Sanghun Park, Gyutae Park, Sehyuk Oh, Yunhwan Park, Yuna Kim, Jaeyoung Kim, Jungseok Choi

**Affiliations:** Department of Animal Science, Chungbuk National University, Cheongju, 28644 Korea

**Keywords:** Cell biology, Molecular biology, Stem cells

## Abstract

The aim of study was to investigate proliferation and differentiation capacities of Hanwoo myosatellite cells for the development of Hanwoo cell cultures. From P1 to P19, the number of live cells decreased and the cell size increased. It was confirmed that the *PAX7* mRNA was higher in P3 than P6 and P9 (*p* < 0.05). The maximum differentiation score was measured from P1 to P12. The maximum differentiation score maintained high from P1 to P10. Immunostaining was performed for both P1 and P10 cells to investigate differentiation characteristics. And there were no significant differences in differentiation characteristics between P1 and P10 cells. *MYOG* mRNA was low, whereas *C-FOS* mRNA was high (*p* < 0.05) in the late passage. Myosin and Tom20 protein also showed low values in the late passage (*p* < 0.05). In conclusion, our results suggest that it is appropriate to use P1 to P10 for the production of cultured meat using Hanwoo muscle cells. If cell culture meat production is performed without differentiation, the passage range may increase further. These results provide basic essential data required for further development of Hanwoo cell cultures, which could provide a valuable source of protein for human populations in the future.

## Introduction

Today’s animal husbandry techniques must mass produce high-quality, inexpensive meat, milk, and eggs through eco-friendly, socially responsible, and economically sustainable production systems^1^. In short, the livestock industry contributes strongly to solving global food security problems, as well as provides economic value and improves national well-being through increased food security and nutrition^2,3^. The world population was 6.9 billion in 2010, 7.7 billion in 2020, and is expected to be 9.1 billion in 2050^3^. Globally, average personal income and population growth are increasing the meat consumption per capita^4^. Even if meat consumption declines in developed countries, global meat consumption will likely continue to rise as developing countries such as China, India, and Russia increase their meat consumption with rising personal incomes^5,6^. As the world population grows, meat production increases to meet the growing demand for meat. The problem here is the steep increase in livestock production. Animal agriculture contributes about 14.5% of global greenhouse emissions and occupies about 77% of the earth’s habitable land to raise and fed livestock while only providing about 17% of global calory supply^7^. Another concern is that climate change issue will affect the sustainability of the livestock industry^8,9^. It will likely include decreased pasture productivity, increased instances of livestock parasites and diseases, and increased competition for land and water^10^. There are many countries vulnerable to future animal protein shortages due to climate change and populations growth^11^. To solve some of the environmental issues that come with livestock production, the livestock industry needs to make meat production more environmentally friendly. Additionally, the livestock industry needs to reduce the water and grain required to produce animal protein^5^. In light of these issues, research into more efficient protein production methods is ongoing and aims to comply with environmental and animal welfare issues while increasing food security^12^. Recently, the term 'cell agriculture' has been proposed for the field of work that focuses on growing cells to be used as food, cosmetics, or ingredients^13^. Cultured meat technology is the in vitro production of cells from certain stem cells to produce proteins and structures similar to animal-based meat^14,15^. If there are appropriate strategies for realizing cultured meat products, they can be a solution to the problems of industrial livestock farming^16,17^. Consumers who want to consume animal protein and are responsible for the state of livestock production are also interested in cultured meat as a sustainable alternative^18–20^. In order for cultured meat to be accepted as a meat substitute and industrialized, technical challenges including regulation, cost, mimic and efficiency must be met^20,21^. One major technical challenge is to produce a product as similar to currently consumed meats as possible. This is done by creating complex structures containing organized myofibers, various fats and bones in cultured meat products^16^. The basic research and developments should be established before the field can make a headway in providing consumers with new cultured meat products^20,21^. It is also important to maintain the ability to proliferate and differentiate cells used in cultured meat production. Hanwoo (Korean cattle) as an indigenous beef cattle are known to have an inferior ability to produce meat because of a low milk producing capacity and slow growth rate, while having a relatively favorable meat quality^22^. Hanwoo is the most popular beef consumed in Korea. Due to the genetic ability of Hanwoo, its deposition of fat is larger than that of European breeds. It is characterized by excellent eating quality and unique scent^23^. Hanwoo cattle has maintained its character as a purebred from the end of 1900 to the present^22^. Hanwoo cattle has undergone long-term artificial selective breeding as part of a national breeding program aimed at improving their meat qualities and characteristics. Genetic improvements of Hanwoo cattle has been carried out using traditional selection methods based on phenotypic information such as the Korean proven bull number (KPN). Although the nature of genes that influence economically important traits is not generally known, artificial selective breeding in this way has been successful. Hanwoo cattle has less diversity and relatively fixed genetic variation than other breeds of cattle^24–26^. Since Hanwoo breeds have relatively less genetic diversity than other small species, the characteristics of cells isolated from Hanwoo muscle tissue will also be relatively small.

Therefore, the purpose of this study was to contribute to the development of cultured meat technology. There are few studies on cultured meat using Hanwoo satellite cells. In this experiment, the proliferation and differentiation of Hanwoo satellite cells was observed through subculture. And the experimental results are expected to become standard data for the production of cultured meat using Hanwoo muscle satellite cells. A range that can be used for the production of cultured meat of the estimated Hanwoo muscle cells was proposed.

## Materials and methods

All animal studies were approved and performed within the guidelines of the Institutional Animal Care and Use Committee (IACUC) of Chungbuk National University, Republic of Korea.

### Primary Hanwoo muscle satellite cell isolation and fluorescence-activated cell sorting (FACS)

The top round muscle tissues of a 33-month-old and 34-month-old Hanwoo (Korean native cattle) steer carcass were collected from FarmStory Hannaeng Central Factory in Cheongwon-gu, Cheongju-si, Chungcheongbuk-do. After storage in an ice box and transport to the lab, the muscle tissues were collected and dissociated with a collagenase type 2 mix (Worthington, Cat # LS004176). The connective tissue and muscle satellite cells were then separated for 5 min at 800 g using a centrifuge. The pellet obtained by centrifugation was filtered with 100um and 40um filters, and red blood cells were lysed with ACK (Ammonium-Chloride-Potassium) lysis buffer. The obtained Hanwoo muscle satellite cells were then finally stored in freezing medium to be stored in liquid nitrogen until the experiment (Cell culture freezing medium, Gibco, Cat # 12648010). These cells were considered passage 0 (P0).

**Figure 1:**
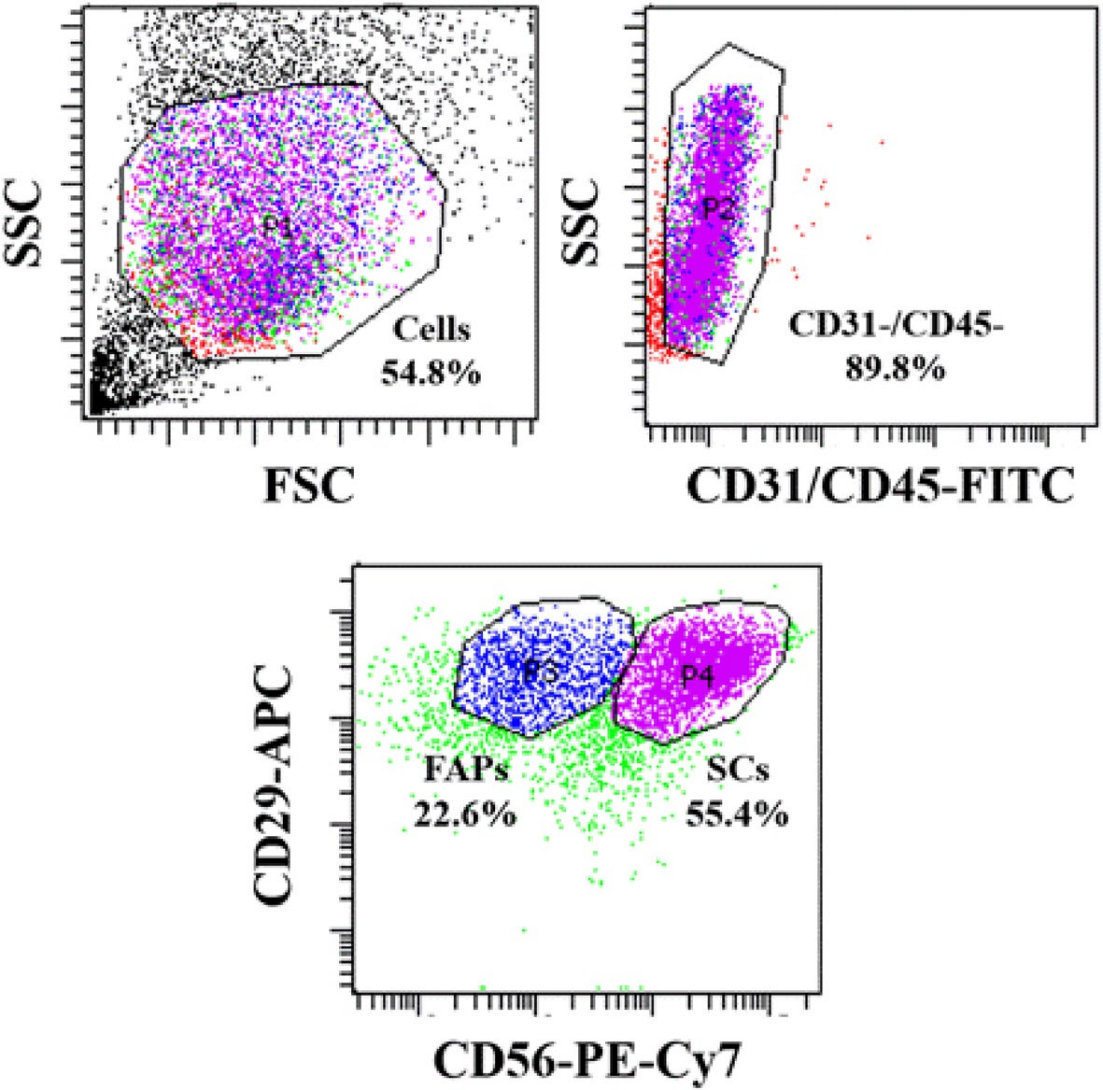
Purification of muscle satellite cells from Hanwoo muscle. Representative flow cytometry plots of unsorted Hanwoo myosatellite cells (SC), based on surface expression of CD31/CD45-FITC, CD29-APC, and CD56-PE-Cy7. Black colored gates indicated FACS stratedgy. Figures denote cells within the respective gates, as a percentage of the parent population.

Prior to FACS, isolated cells were cultured on 5 mg/ml bovine collagen type I (Sigma-Aldrich, Cat # C2124) pre-coated flasks until confluent and the cells were transferred on FACS buffer (1:100, Bovine Serum Albumin in PBS(Phosphate buffered saline)) and stained with APC anti-human CD29 Antibody (1:10, BioLegend, Cat # 303008), PE-CyTM7 anti-human CD56 (1:10, BD, Cat # 335826), FITC anti-sheep CD31 (1:10, BIO-RAD, Cat # MCA1097F), FITC anti-sheep CD45 (1:10, BIO-RAD, Cat # MCA2220F) for 30 min in 4 °C. After antibody incubation, the cells were washed twice with cold PBS and reconstituted in GM (growth medium, 20% fetal bovine serum (FBS, Gibco, Cat # 16000044), 1% PSA (penicillin–streptomycin–amphotericin B solution, Lonza, Swiss, Cat # 17745E), 5 ng/ml recombinant human fibroblast growth factor (rhFGF(basic), Promega, Cat # G5071)) in Ham’s F-10 nutrient mix (Gibco, Cat # 11550043)). The CD31−, CD45−, CD29+, CD56+ cells were sorted by using FACS Aria II Cell Sorter (BD) (Fig. 1)^27^.

### Satellite cell proliferation culture and myogenic differentiation

The flasks were coated with 0.05% bovine collagen type I (sigma) for proliferation culture and coated with Matrigel (Corning) for differentiation culture. After thawing the Hanwoo muscle satellite cells, they were cultured on collagen coated flasks in GM and cultured on Matrigel coated flasks in differentiation medium (DM, DMEM-Dulbecco’s Modified Eagle Medium (Gibco, Cat # 11995065), 2% FBS, 1% PSA). The cells were stained with trypan blue for measurement via automated cell counter (Countess, Invitrogen). Hanwoo satellite cells were then seeded 1800 live cells/cm^2^ on flasks and these were placed into an incubator at 37 °C, 5% CO_2_. These cells were set passage 1 (P1). After 24 h, the P1 cells were set to proliferation day 1 and incubated at 37 °C, 5% CO_2_ until 6th day.

### Subculturing of Hanwoo muscle cells

On 6th day of P1 Hanwoo cells, the growth medium was removed from flask and the flask was washed with PBS before being treated with trypsin–EDTA to detach the cells and make a cell suspension. The cell suspension was then neutralized with TNS (Trypsin Neutralizer Solution, 2% FBS in PBS), centrifuged at 350 g for 5 min, and supernatant was removed. After staining the cells with trypan blue, the number of living cells was counted using an automated cell counter (Countess, Invitrogen). Cells were then reattached by seeding 1800 live cells/cm^2^ into newly prepared coated flasks and the cells in those flasks were considered to be in their second passage (P2). This process proceeded to P19. For the differentiation phases, the growth medium was replaced with the differentiation medium on day 6. After 24 h, those cells were marked as differentiation day 1 and they were allowed to incubate for a full 6 days at 37 °C, 5% CO_2_.

### Differentiation image analysis from brightfield microscopy

For each passage, brightfield images were captured every 24 h to create for 6 days differentiation measurements. Images were scored by six independent observers based on a scoring system ranging from 0 (no differentiation) to 6 (high myotube area). Representative brightfield microscopy images became the criterion for the maximum differentiation score^28^.

### Immunofluorescence staining

Cultured satellite cells were fixed with cold 2% paraformaldehyde (in PBS) for 45 min, rinsed with PBS, and permeabilized in 0.5% Triton X-100 (in PBS) for 20 min. The permeabilized cells were then incubated with a Mouse Monoclonal Anti-Myosin antibody (1:100, Sigma, Cat # M4276) in 2% BSA (bovine serum albumin, in PBS) overnight at 4 °C. After washing with 0.05% Tween 20 (in PBS), cells were incubated with Alexa Fluor 488 labeled goat anti-mouse IgG1 (1:200, Invitrogen, Cat # A-21121) for 30 min at room temperature and washed 0.05% Tween 20 (in PBS). Cells were then treated with Hoechst 33,342 for 2 min at room temperature (1:2000, Invitrogen, Cat # H3570). Immunofluorescence staining was performed on P1 and P10 cultured cells of differentiation day 4 in 96 well plates. All images were measured by trained analysts using the ImageJ program (NIH, Bethesda, MD, USA).

### Images acquisition and analysis

**Figure 2:**
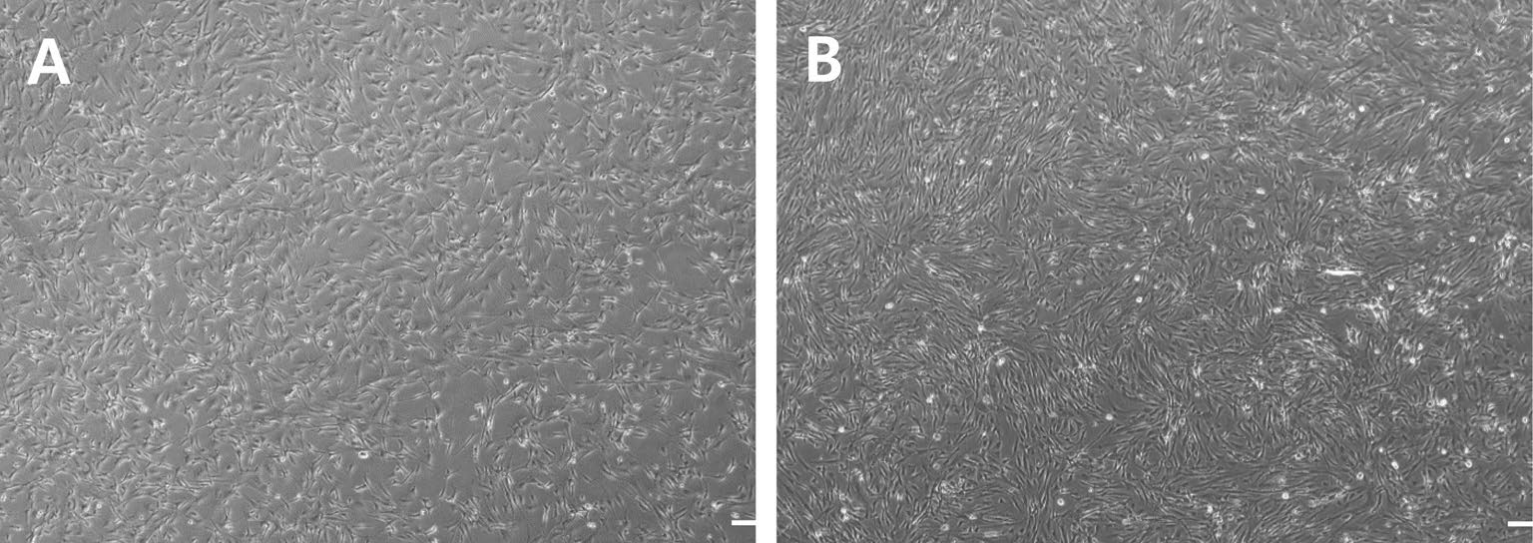
Representative confluent images of Hanwoo myosatellite cells. Images of Hanwoo myosatellite cell confluent on flask. Figure 1A is proliferation day 5 (80% confluence) of Hanwoo myosatellite cell, Fig. 1B is proliferation day 6 (90% confluence) of Hanwoo myosatellite cell. The images were taken at 40 × magnification. Scale bar = 100 µm.

**Figure 3:**
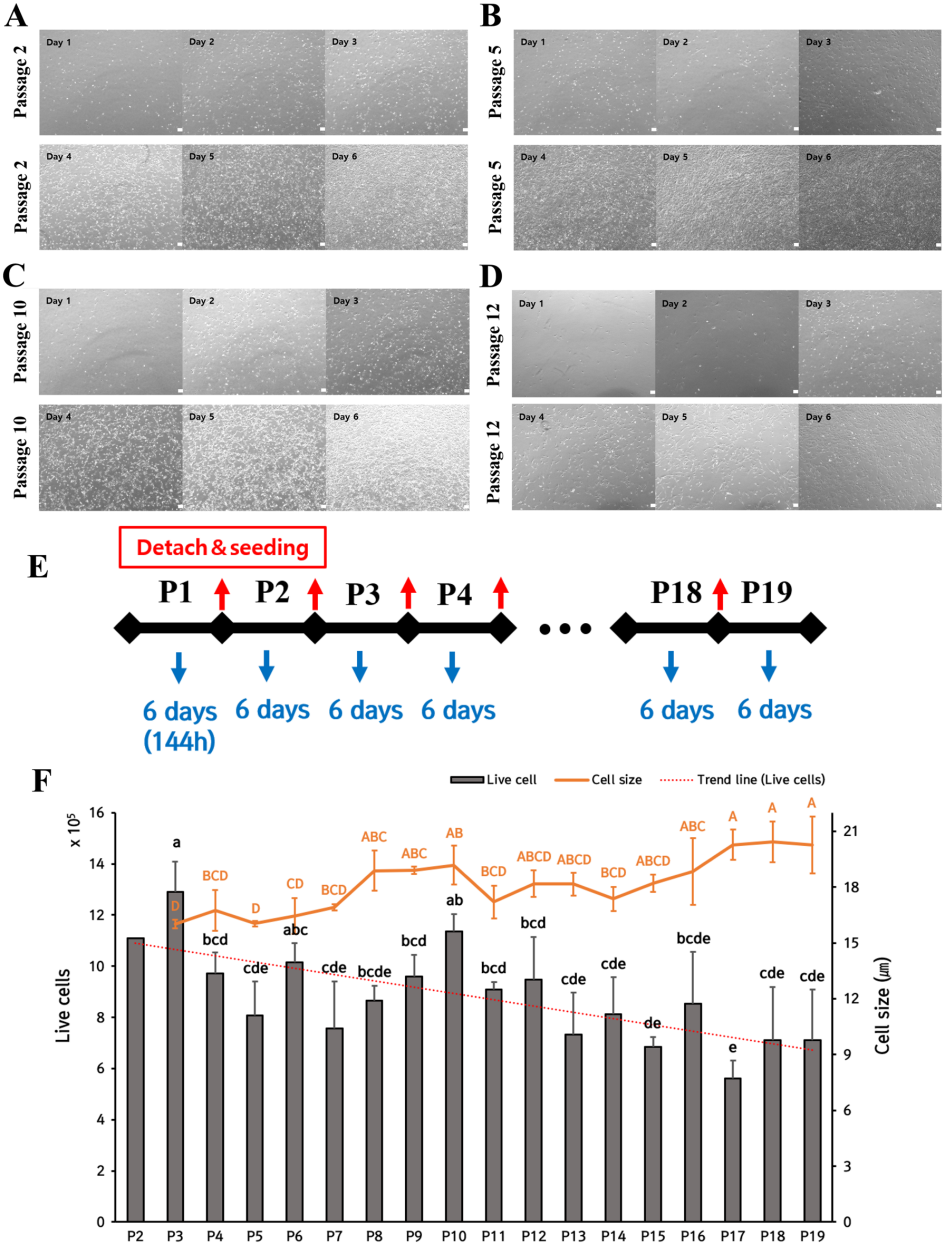
Images of proliferation subculture of Hanwoo muscle cells, figure of culture period, live cells count, and cell size. Proliferation of cultured Hanwoo myosatellite cells from day 1 to day 6. Hanwoo satellite cells were cultured at 37 °C with 5% CO_2_ in collagen coated flasks. Brightfield microscopy images (**A**–**D**) showed the proliferation from day 1 to day 6 from P2, P5, P10, and P12. These images were taken at 40 × magnification. (**E**) Figure of proliferation culture period. (**F**) Number of live cells, trend line, and cell size (µm) of live cells on the 6th day of culture of Hanwoo satellite from P3 to P19 (n = 3). The number of live cells and cell size of P2 were not statistically processed because there was no statistical repetition. Black lower-case letters were superscripts of live cells, and orange upper-case letters were superscripts of cell size. Live cell counting and cell size data were presented as mean ± standard deviation. Mean ± standard deviation with differing letters differs significantly (*p* < 0.05). Scale bar = 100 µm.

The proliferation and differentiation of Hanwoo muscle cells in all passages were observed with the EVOS M5000 imaging system (EVOS M5000, Invitrogen, Cat # AMF5000) (Figs. 2, 3, 5). Four images of stained p1 and p10 cells were randomly acquired (Fig. 6) and the average width of myotubes (myotube width (μm)), the number of myotubes, myotube area ratio on the image (myotube area (%)), the number of nuclei, nucleus area ratio on the image (nucleus area (%)), and the fusion index (%) were measured by ImageJ (NIH, Bethesda, MD, United States)^29^. Myotubes were classified as elongated structures containing three or more nuclei within a single membrane structure. The number of times the width of myotubes was measured was the number of myotubes. The width of myotubes were measured both at the beginning of relatively thick myotubes, as well as at the beginning of the relatively thin myotubes that split from the thick myotubes on the image. The fusion index was calculated as the number of nuclei incorporated into myotubes expressed as a percentage of the total number of nuclei in the image frame^30,31^.

### Western blot analysis

Hanwoo muscle cells cultured for 4 days from P3, P6, P9 and P12 were collected in sodium dodecyl sulfate (SDS) sample buffer and heated for 5 min at 95 °C. Proteins of the cells were separated by SDS‐polyacrylamide gel electrophoresis and electrically transferred onto polyvinylidene fluoride (PVDF) membranes. The transferred membranes were blocked in EveryBlot Blocking buffer (Bio-Rad, Cat # BR12010020, USA) for 1 h and then incubated overnight at 4 °C with primary antibodies. The primary antibodies used were monoclonal anti-β-actin antibody produced in mice (1:3000, Sigma-Aldrich, Cat # A2228), myosin 4 monoclonal antibody (1:1000, Invitrogen, Cat # 14-6503-82), Tom20 (F-10) (1;1000, Santa Cruz, Cat # sc-17764). After washing three times for 10 min with TBST (tris-buffered saline with 0.1% tween 20), the membranes were incubated for 1 h at room temperature with affinity-purified goat anti-mouse IgG (H + L) horseradish peroxidase (HRP)-conjugated secondary antibody (1:3000, Bio-Rad, Cat # BR1706516). The membranes stained with antibodies were exposed to Clarity Western ECL Substrate (Bio-Rad). Blots were visualized using a charge-coupled device (CCD) camera and IQ800 control software (Cytiva)^32^.

### Real-time reverse transcription (RT)–quantitative PCR

The cultured cells were collected, and total RNA was extracted using the Total RNA Extraction Kit (iNtRON Biotechnology, Cat # 17221, Korea) according to the manufacturer’s instructions. cDNA was obtained using the High Capacity cDNA Reverse Transcription Kit (Thermo Fisher, Cat # 4368814). Real-time quantitative PCR was performed using Fast qPCR 2 × SYBR Green Master Mix (Cat # EBT-1821). Amplification was conducted as follows: 50 °C for 2 min and 95 °C for 10 min, followed by 40 cycles of 95 °C for 15 s, 52–55 °C for 1 min. The target genes were *PAX7, MYOD, C-FOS, MYOG,* and *GAPDH*^32^. The primers used to amplify each gene were shown in Table 1. The mRNA quantities were analyzed using the 2−ΔΔCT method^33^.

**Table 1:** Sequences of the primers used in the RT-qPCR study.

Primer	
*PAX7*	F: CTCCCTCTGAAGCGTAAGCA
	R: GGGTAGTGGGTCCTCTCGAA
*MYOD*	F: GCAACAGCGGACGACTTCTA
	R: AGGGAAGTGCGAGTGTTCCT
*C-FOS*	F: CTGAGCCCTTTGATGACTAC
	R: GACGAAGGAAGACGTGTAAG
*MYOG*	F: AGAAGGTGAATGAAGCCTTCGA
	R: GCAGGCGCTCTATGTACTGGAT
*GAPDH*	F: CACCACCATGGAGAAGGCCG
	R: GAACACGGAAGGCCATGCCA

### Statistical analysis

For statistical analysis, data from at least three technical replicates were analyzed. Statistical analysis was conducted with one-way analysis of variance (ANOVA) using the General Linear Model (GLM) procedure of the SAS program (Statistical Analysis System 2002, Cary, NC, USA), and the significance of the comparisons between the means of the treatment groups was verified (*p* < 0.05) using Duncan’s multiple range test.

### Ethics approval

The animal study protocol was approved by the Chungbuk National University Institutional Animal Care and Use Committees.

## Results

### Maintaining the proliferation capacity of Hanwoo myosatellite cells

Figure 2 shows the degree of confluence at day 5 and day 6 as a percentage of total coverage on P19. Anchorage-dependent cell lines growing in monolayers need to be subculture at regular intervals to maintain exponential growth. When anchorage-dependent cells are approximately 70–90% confluent, they are ready to be subculture. Cell lines that grow attached to the surface should be subculture regularly before the confluent period (the period when growth is stagnant) to ensure viability and genetic stability^34^. In this experiment, all passages were 80% confluent on day 5 and 90% on the day 6, and it was considered appropriate to subculture between day 5 and 6 (Fig. 2). In order to obtain a sufficient number of cells, subcultures were performed only once the confluence reached 90%^35^.

Hanwoo myosatellite cells were cultured for a total of 6 days between each passage (Fig. 3E). On day 1, it was confirmed that anchorage dependent Hanwoo myosatellite cells did attach to the flask. On day 6 of culture, Hanwoo myosatellite cells were confluent up to 80–90% for all passages with which the experiment was conducted. In this experiment, the same subculture method was performed for all passages in order to maintain constant proliferation of Hanwoo myosatellite cells. Image data were gathered for all passages to determine the overall trend. Figure 3A–3D are representative image data. It shows the proliferation trend of P2, P5, and P10 among subcultures from P2 to P19. Among passages surveyed, the highest number of cells was found in P3 (Fig. 3F). The trend line of live cells tended to decrease gradually from the early passage to the late passage (until P19). It was confirmed that the proliferation potential decreased as the subculture progressed. In contrast, the cell size increased (Fig. 3F).

**Figure 4:**
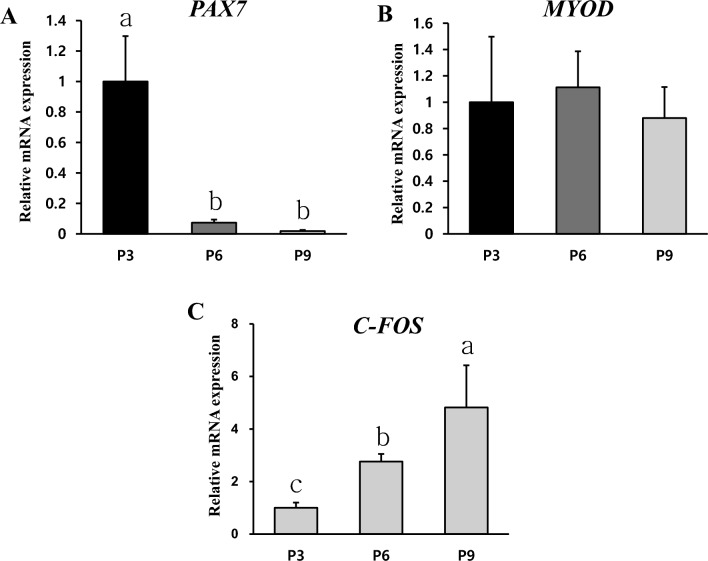
Relative proliferation mRNA levels of Hanwoo muscle cells. Relative PAX7 and MYOD mRNA levels of Hanwoo myosatellite cells cultured for 6 days were compared among P3, P6, and P9. *PAX7* (**A**), *MYOD* (**B**) and *C-FOS* (**C**) mRNA levels were analyzed. *GAPDH* was used as an internal control for RT-PCR. Experiments were performed repeated three times (n = 3). Mean ± standard deviation with differing letters differs significantly (*p* < 0.05).

The proliferation potential was confirmed through biomarkers related to the proliferation of early (P3), middle (P6), and late passage (P9). It was confirmed that the *PAX7* mRNA level was higher in P3 than in P6 and P9 (Fig. 4A) (*p* < 0.05). It suggests that the proliferation potential was confirmed through biomarkers related to proliferation of early, middle, and late passages. *PAX7* mRNA levels decreased significantly at late passage compared to those at early passage (Fig. 4A), suggesting that quiescent satellite cells were significantly reduced during the subculturing process. The difference in the number of stationary satellite cells between early passage (P3) and late passages (P6 and P9) was expected to be large. *MYOD* mRNA levels remained unchanged from P3 to P9 (Fig. 4B). From early passage to P9, differentiation potential was expected. The increase in *C-FOS* mRNA from P3 to P9 appeared to be related to the maintenance of live cells until late passage (Fig. 4C). Downregulation of *C-FOS* mRNA is essential for terminal differentiation and myotube formation^36,37^.

### Measurement the differentiation capacity of Hanwoo myosatellite cells

Major components of Matrigel, an ECM used for the differentiation of various cells, were found to be laminin and enactin, which share relatively few common peptides with collagen^38^. It is known that the growth capacity of cells in Matrigel is higher than that in collagen and that α7β1 binding sites in Matrigel are related to differentiation^39^. Unlike collagen coating in a proliferative culture, this experiment used Matrigel as an ECM for observing the differentiation of Hanwoo myosatellite cells. The presence of myosin is often considered evidence that muscle cells have differentiated. This can be confirmed through immunofluorescence analysis^40^. Previous studies have demonstrated that serum can promote the growth of muscle stem cells, but inhibit muscle differentiation at high concentrations^41^. In this experiment, FBS was mixed with the differentiation medium at a smaller ratio than the growth medium in order to prevent inhibition of muscle cell differentiation.

**Figure 5:**
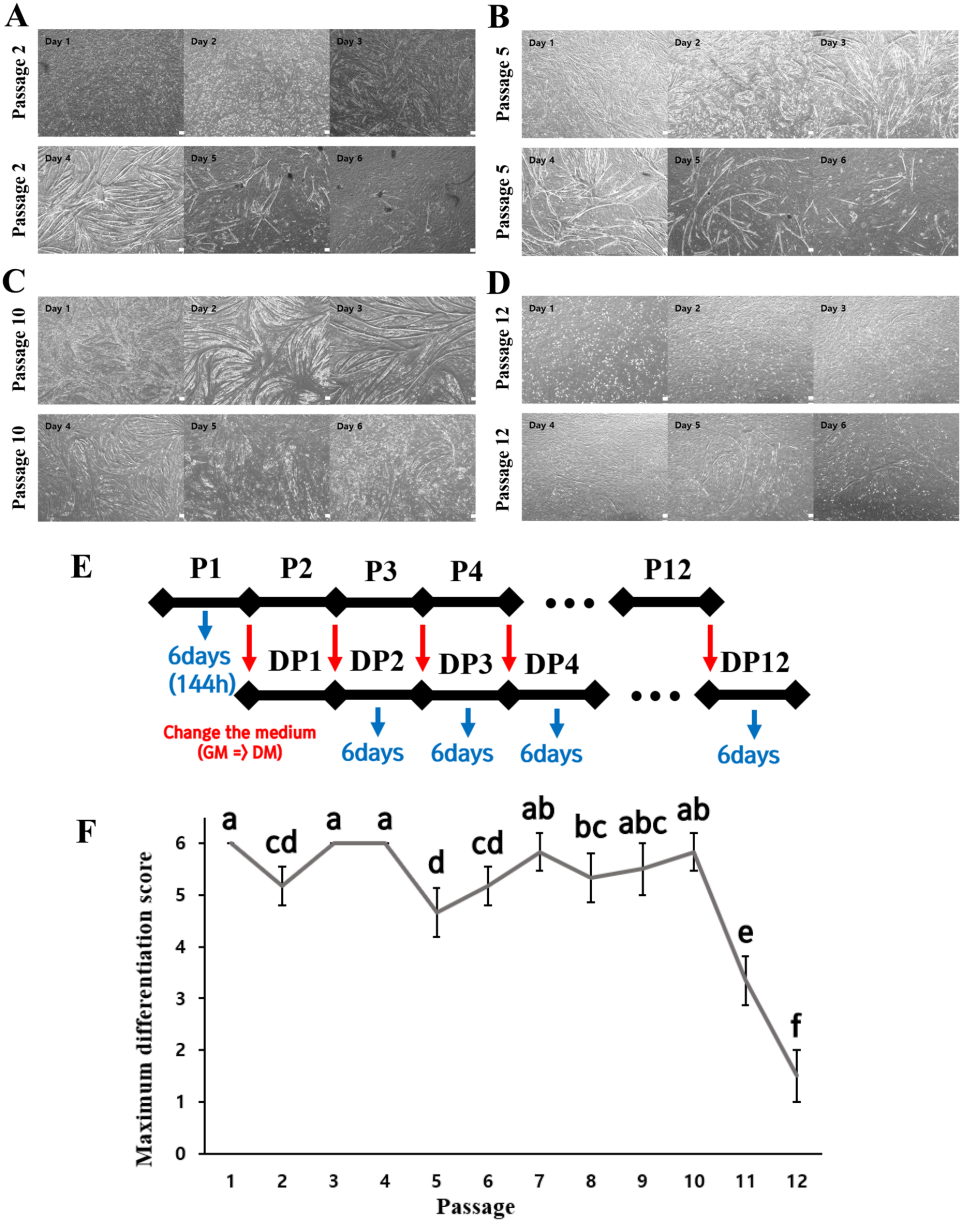
Images of differentiation subculture of Hanwoo muscle cells, figure of culture period, and maximum differentiation score. Differentiation of cultured Hanwoo myosatellite cells from differentiation culture day 1 to day 6. Hanwoo satellite cells were cultured at 37 °C with 5% CO_2_ in Matrigel coated flasks. Brightfield microscopy images (**A**–**D**) showed the differentiation from day 1 to day 6 from P2, P5, P10, and P12. These images were taken at 40 × magnification. (**E**) was a figure of differentiation culture period. (**F**) Maximum extent of differentiation over long-term proliferation based on visual differentiation score from day 1 to 6 by six independent observers (κ = 0.552) of brightfield microscopy images. Mean ± standard deviation with differing letters differs significantly (*p* < 0.05). Scale bar = 100 µm.

Differentiation phases were monitored for 6 days (Fig. 5E). On differentiation day 1, sufficient cells proliferated, and differentiation started. It was confirmed that most myotubes were formed between 2 and 4 days of differentiation. Our data clearly showed that differentiated myotubes detached on differentiation day 5–day 6 (Fig. 5A–D). Hanwoo satellite cells were subculture from P1 to P12 in order to observe the differentiation level by passage. The formation of myotube decreased significantly after P12. Thus, differentiation culture was not performed for subsequent passages (Fig. 5E,F). Image data were acquired during each passage to better determine the overall trend. Figure 5 is a representative image showing differentiation trend of P2, 5, 10, and 12 among subcultures from P1 to P12. Figure 5F shows results of the maximum differentiation score. Differentiation and myotube formation scores of Hanwoo myosatellite cells dropped significantly at P11 and P12 compared to P10.

**Figure 6:**
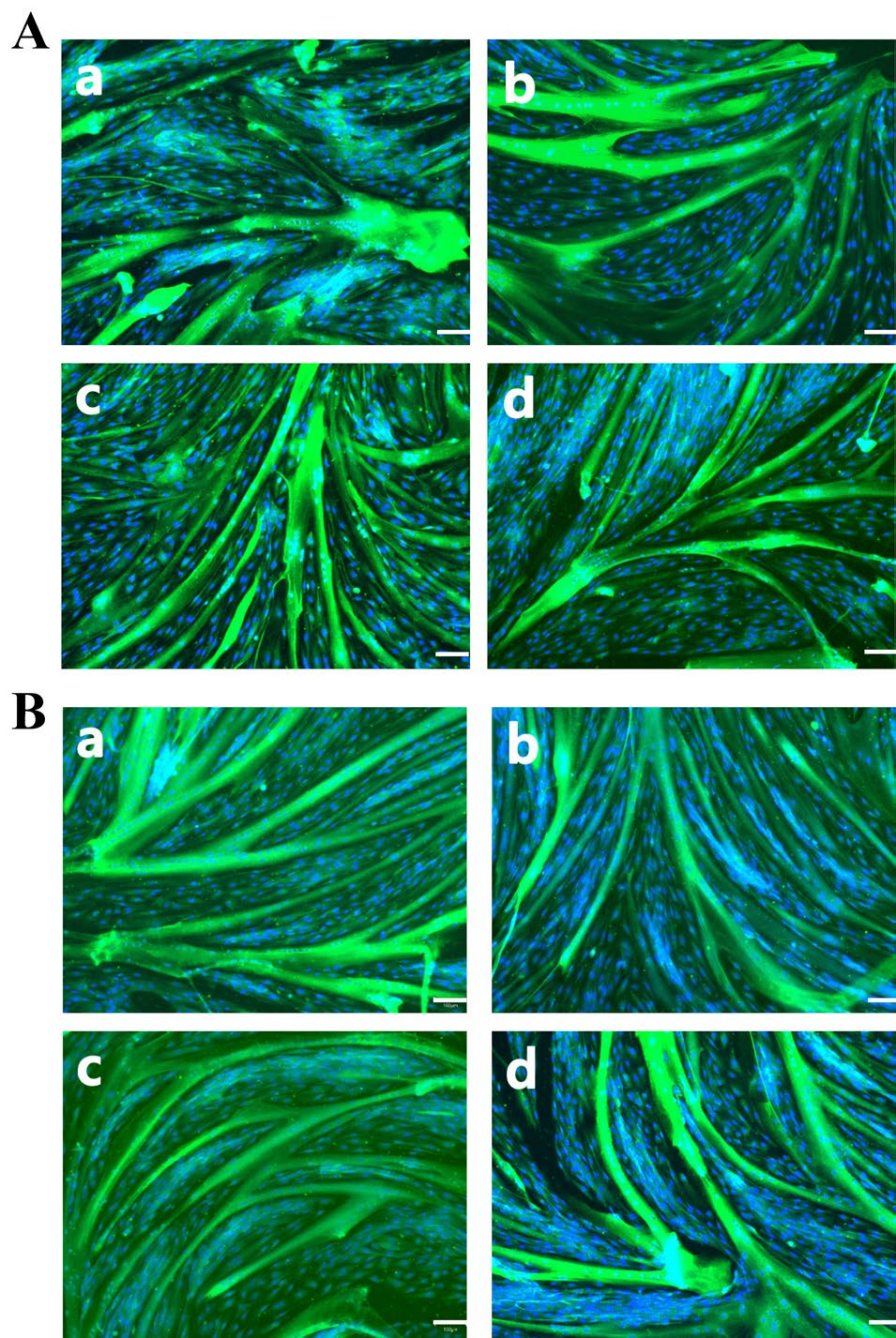
Immunofluorescence staining of Hanwoo muscle cell at P1 and 10. Immunofluorescence staining of Hanwoo myosin protein in myotube was performed at differentiation day 4 for P1 and P10. (**A**) (a, b, c, d) taken at random (at the top, bottom, left and right points, respectively) at P1 and (**B**) (a, b, c, d) taken at random (at the top, bottom, left and right points, respectively) at P10. Images were taken at 100 × magnification. Scale bar = 100 µm.

Figure 6 shows images of myosin and nucleus fluorescence staining of Hanwoo skeletal muscle cells. To characterize morphological changes, myotubes are often quantified via immunofluorescence-staining of myotube cytoskeletal markers (e.g., desmin or myosin heavy chain) along with DAPI^42,43^. The diameter, surface area, and nuclear fusion index (defined as the number of nuclei incorporated into myotubes, expressed as a proportion of the total visible nuclei in each field of view) of myotubes could be measured manually using a public-domain software such as ImageJ^44^. Capacities for differentiation of cells at P1 and P10 are shown in Fig. 6. Table 2 shows results of comparing differentiation characteristics of cells at P1 and P10. There were no significant differences in myotube width, myotube area, number of nuclei, or nucleus area between passages. The fact that there were no significant differences in Hanwoo muscle differentiation phenotype characteristics between P1 and P10 suggested that the differentiation capacity was not significantly lost based on the expression of myotubes throughout passages from 1 to 10.

**Table 2:** Hanwoo cell differentiation characteristics.

	Number of myotube	Myotube thickness (μm)	Myotube area (%)	Number of nucleus	Nucleus area (%)	Fusion index (%)
P1	77	55.01 ± 32.23	30.00 ± 9.44	867.00 ± 164.68	11.88 ± 4.77	33.30 ± 6.46
P10	80	54.12 ± 24.05	34.12 ± 11.33	983.75 ± 228.77	13.76 ± 2.28	37.41 ± 12.87

Myotube width was analyzed for 77 myotubes in p1 and 80 in p10. The rest of the values were analyzed from cells in Fig. 6p1(a,b,c,d) or p10(a,b,c,d). All images were measured by trained analysts using ImageJ. There were no significant differences between the groups in these data.

**Figure 7:**
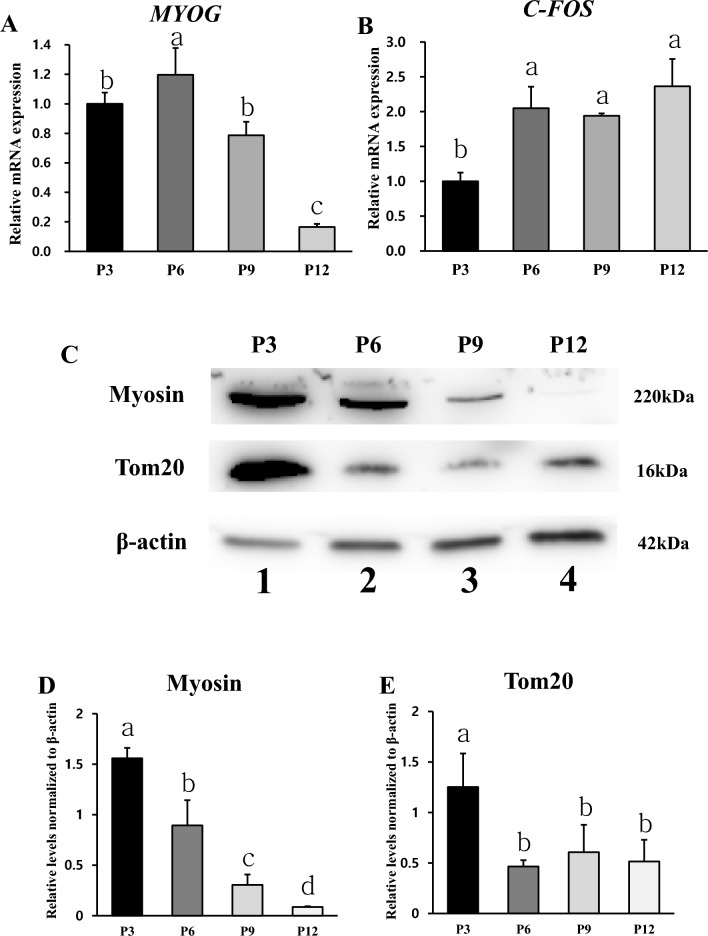
Relative differentiation mRNA and protein levels of Hanwoo muscle cells. Relative mRNA and protein expression levels in Hanwoo muscle cells differentiated for 4 days at P3, P6, P9, and P12. Relative *MYOG* (**A**) and *C-FOS* (**B**) mRNA levels were analyzed. *GAPDH* was used as an internal control for RT-PCR. Experiments were repeated three times (n = 3). Mean ± standard deviation with differing letters differs significantly (*p* < 0.05). Representative images (**C**) of western blotting against Myosin, Tom20, and β-actin using lysates of differentiated Hanwoo muscle cells at P3 (Lane 1), P6 (Lane 2), P9 (Lane 3), P12 (Lane 4). (**D**) and (**E**) are relative Myosin and Tom20 levels normalized to β-actin from (**C**). Mean ± standard deviation with differing letters differs significantly (*p* < 0.05).

Figure 7A,B shows *MYOG* and *C-FOS* mRNA levels in Hanwoo muscle cells differentiated for 4 days at P3, P6, P9, and P12. At P3, P6, and P9, *MYOG* mRNA level was significantly higher than that at P12 (Fig. 7A). The amount of *C-FOS* mRNA, a transcription factor for promoting proliferation, was higher at P6, P9, and P12 than that at P3 (Fig. 7B). Figures 6E and 7C,D shows protein expression levels and band of Myosin and Tom20 in Hanwoo muscle cells differentiated for 4 days at P3, P6, P9, and P12. Myosin protein expression level decreased significantly as passages increased (Fig. 7D). Tom20 protein level was the highest in P3. There was no difference of Tom20 protein levels in P6, P9 and P12 (Fig. 7E). The number of mitochondria related to skeletal muscle protein and energy metabolism decreased as late passage progressed.

## Discussion

Most of cells used in the production of cultured meat are adult stem cells and primary cells isolated from livestock. The proliferation potential and differentiation potential are important requirements for producing cultured meat because of finite cell replication. This senescence is mediated by the shortening of chromosomal telomeres that occurs along with cell division^45^. In addition, it has been reported that muscle stem cells cultured in vitro can gradually lose stemness due to a lack of signaling molecules^46^. Subculturing is a good method to identify the potential of cell proliferation and differentiation by causing continuous cell replication. In general, proliferation and myotube formation decrease as the passage number increases^46^. This might be related to replicative senescence cells^47^. In addition to previous studies, by measuring the number and size of live cells at each passage in this experiment, it can be inferred that cell senescence progressed in the later passages through a decrease in proliferation capacity and an increase in cell size. The decrease in *PAX7* mRNA level after passage 3 indicated a decrease in quiescent satellite cells, which meant that the growth potential of Hanwoo muscle cells had decreased. At P3, P6, and P9, there is no significant difference in the amount of *MYOD* mRNA level known to affect differentiation. Thus, the reduction in differentiation capacity could not be explained by difference in *MYOD* mRNA level. Although *MYOD* is an important factor for muscle growth, there was no significant difference even when the passage increased in vitro culture. As a result of this experiment, the decrease in proliferative capacity in the latter passage is not considered to be related to the change in the relative amount of *MYOD* mRNA. Myotubes are multinucleate cell produced by the differentiation of myoblasts into myocytes and the fusion of several clusters. Myotubes have rows of centrally located nuclei and peripheral masses of forming contractile myofilaments and cytoskeleton that soon become oriented into sarcomeres and myofibrils with restoration of cross-striations in the immature myofibers^48^. Hanwoo muscle cells maintained myotube phenotype and characteristics of early passage until P10. There was no significant difference in myotube morphology or fusion index between P1 and P10. The maximum differentiation score remained high until P10. These indicate that the maximum differentiation score can be used to determine the range of available Hanwoo muscle cells for efficient cultured meat production. In addition, the rapid differentiation capacity and myotube formation were steadily maintained in the late passage, and then there was a rapid decrease at a specific point. The c-fos, a transcription factor known to promote proliferation, must be down-regulated to induce differentiation of myocytes. In order to differentiate muscle cells, proliferation inhibition must be performed as a prerequisite. Downregulation of *C-FOS* mRNA is essential for terminal differentiation and myotube formation^36,37^. The fact that *C-FOS* mRNA was not reduced in the late passage might affect the differentiation potential. The *C-FOS* mRNA level was the lowest at P3, indicating that differentiation and myotube formation in Hanwoo cells were higher at P3 than those at later passage. Myogenin is known to affect myotube formation by inhibiting myoblast proliferation^37^. It was confirmed that *MYOG* mRNA decreased in the late passage. Myosin protein values of Figs. 6 and 7D were slightly different in both experiments. In the phenotype analysis of protein through staining, it was determined that myosin maintained skeletal formation up to p10, but it does not appear to be measured the amount of protein. Phenotype analysis by protein staining and protein amount measurement seem to have different meanings. Judging by the results, phenotype analysis by protein staining confirms the skeleton muscle structure and formation characteristics, and protein amount analysis by western blotting measures the density of myosin protein. Therefore, it is believed that there was a difference in the tendency of the two experiments. The mitochondria density in myotube, which is muscle contraction, is higher than myoblasts^49,50^. Therefore, the amount of Tom20 protein, which is mitochondrial protein, became an indicator of relative myotube and differentiation. The differentiation capacity of Hanwoo muscle cells was confirmed through Myosin and Tom20 protein expression. Decreases of myotube cytoskeletal and mitochondrial protein levels of Hanwoo cells in the late passage meant decreased differentiation capacity. It is judged that muscle cell transcriptional regulatory factor of proliferation and differentiation comprehensively decreased differentiation capacity at a specific point. Due to the low genetic diversity of Hanwoo, the common tendency of Hanwoo muscle cells may be that their proliferation and differentiation capacity rapidly decreases at certain points. If these points are confirmed through more advanced studies, there is a possibility that the culture period of Hanwoo muscle cells for the production of cultured meat may be easily defined.

## Conclusion

Using primary cells for cultured meat production is free from genetic recombination and ethical issues as food. For the production and commercialization of cultured meat, research on the utilization of livestock primary cells is essential. In this experiment, Hanwoo myosatellite cells showed a trend of decreasing cell counts at late passage. It is judged that muscle cell transcriptional regulatory factor of proliferation and differentiation comprehensively decreased differentiation capacity at a specific point. Myotube phenotype of Hanwoo cells had no difference between P1 and P10. The maximum differentiation score remained high levels from P1 to P10. The amount of myotube formation and differentiation capacity decreased at later passage. Based on the results, Hanwoo muscle cells measured in this experiment are expected to significantly decrease the productivity of cultured meat after P10. It is judged that it is appropriate to use cells up to P10 when differentiated cells are used to produce cultured meat of Hanwoo muscle satellite cells used in this experiment. The results of this experiment are limited to two Hanwoo muscle cells. If Hanwoo muscle cells of another individual are used in experiments, the differentiation capacity may not be maintained up to P10. However, since the genetic diversity of Hanwoo is relatively low compared to other cattle breeds, similar trends can be inferred in other Hanwoo muscle cells. It is believed that the tendency of proliferation and differentiation capacity to gradually decrease is similar. If the differentiation process of Hanwoo myosatellite cells is omitted to produce cultured meat, the range of passages available will be higher.

### Supplementary Information

Supplementary Information.

## Data Availability

The data presented in this study are available in the current article.
