# Supplementary figures and images for: Investigating proliferation and differentiation capacities of Hanwoo steer myosatellite cells at different passages for developing cell-cultured meat
Source: Sci Rep. 2023 Sep 20;13:15614. doi: 10.1038/s41598-023-40800-7 (PMC10511522; doi:10.1038/s41598-023-40800-7)

**C**

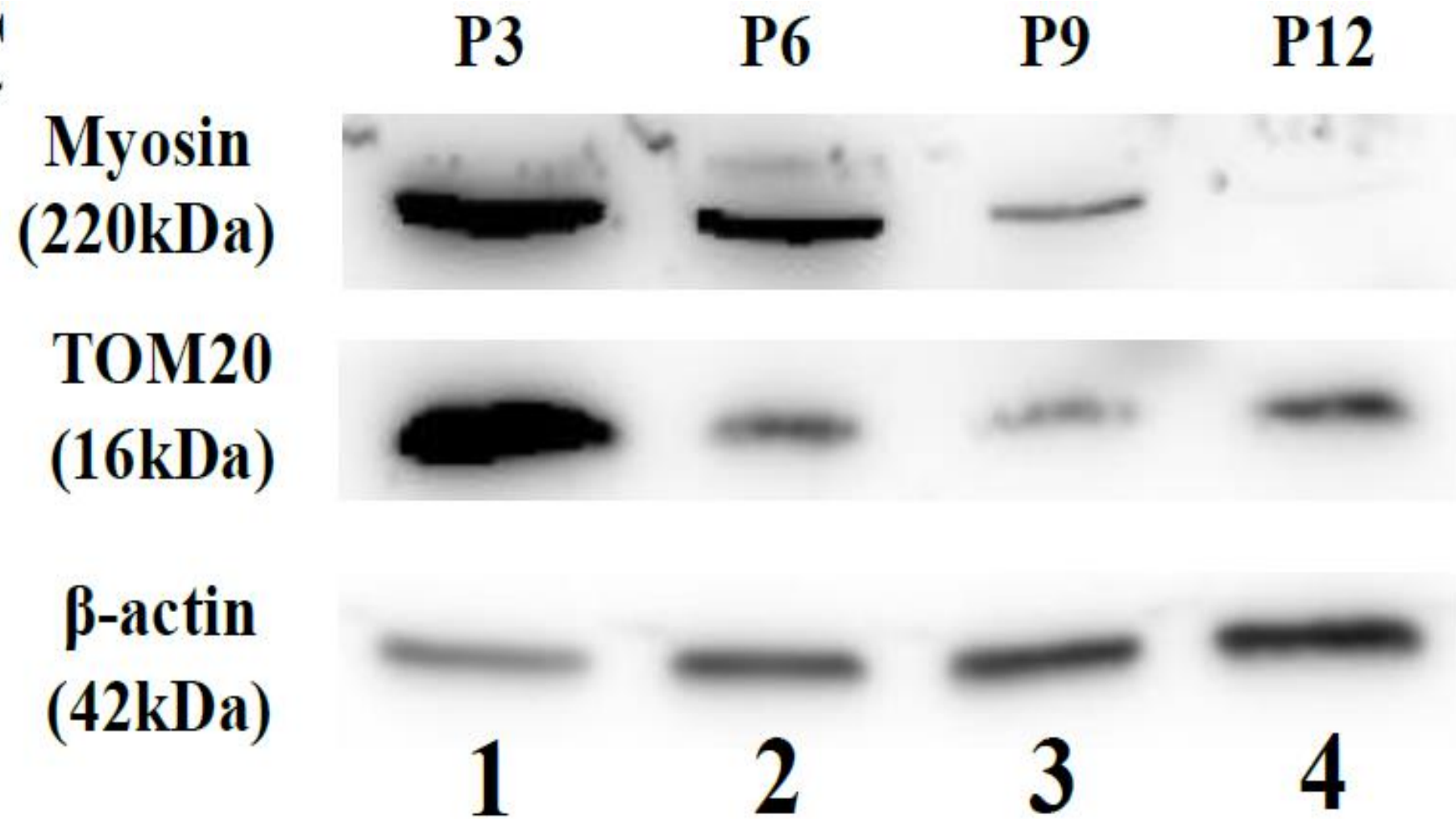

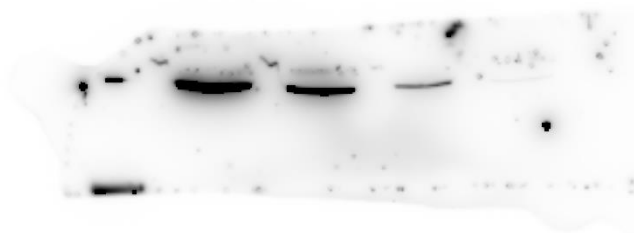

P3,6,9,12 Myosin blots

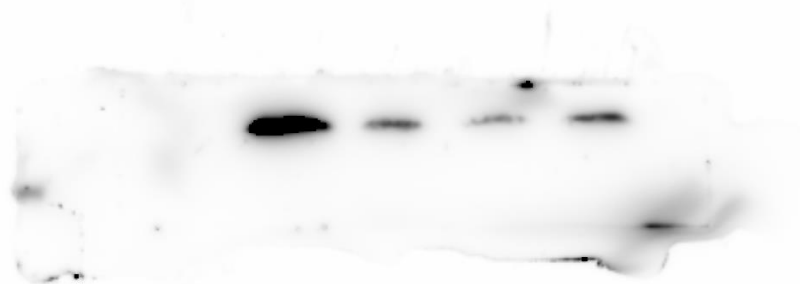

P3,6,9,12 Tom20 blots

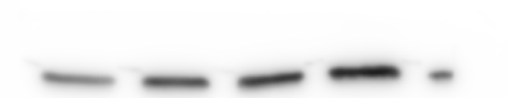

P3,6,9,12 beta-actin blots

Supplement: Supplementary file 1 — Supplementary Information. [file 41598_2023_40800_MOESM1_ESM.pdf]
